# Reconsolidation-Extinction Interactions in Fear Memory Attenuation: The Role of Inter-Trial Interval Variability

**DOI:** 10.3389/fnbeh.2017.00002

**Published:** 2017-01-24

**Authors:** Allison Auchter, Lawrence K. Cormack, Yael Niv, Francisco Gonzalez-Lima, Marie H. Monfils

**Affiliations:** ^1^Department of Psychology, Institute for Neuroscience, University of Texas at AustinAustin, TX, USA; ^2^Department of Psychology and Princeton Neuroscience Institute, Princeton UniversityPrinceton, NJ, USA

**Keywords:** reconsolidation, extinction, fear attenuation, inter-trial interval, retrieval, memory reactivation

## Abstract

Fear extinction typically results in the formation of a new inhibitory memory that suppresses the original conditioned response. Evidence also suggests that extinction training during a retrieval-induced labile period results in integration of the extinction memory into the original fear memory, rendering the fear memory less susceptible to reinstatement. Here we investigated the parameters by which the retrieval-extinction paradigm was most effective in memory updating. Specifically, we manipulated the inter-trial intervals (ITIs) between conditional stimulus (CS) presentations during extinction, examining how having interval lengths with different degrees of variability affected the strength of memory updating. We showed that randomizing the ITI of CS presentations during extinction led to less return of fear via reinstatement than extinction with a fixed ITI. Subjects who received variable ITIs during extinction also showed higher freezing during the ITI, indicating that the randomization of CS presentations led to a higher general reactivity during extinction, which may be one potential mechanism for memory updating.

## Introduction

Current conceptualization of learning and memory has transitioned away from the once dogmatic view that memories are plastic during formation but stable once established (Martin et al., 2000; Dudai, 2004). It is now firmly established that memory retrieval and maintenance are also active processes. Specifically, memory retrieval is a complex process whereby upon recollection, the original memory can enter into a destabilized state, and maintenance of the memory requires restabilization by means of reconsolidation (Misanin et al., 1968; Nader et al., 2000a,b; Nader and Einarsson, 2010). Evidence from several species suggests that reconsolidation blockade via pharmacological (Nader et al., 2000a; Alberini, 2005; Debiec and LeDoux, 2013) or behavioral (Monfils et al., 2009; Schiller et al., 2010; Jones and Monfils, 2013; Luo et al., 2015) interventions during a specific post-retrieval reconsolidation window alters the original memory. The ability to update memories after they have been retrieved has tremendous clinical relevance for treatment of fear and anxiety disorders (Monfils et al., 2009; Rosas-Vidal et al., 2015; Careaga et al., 2016).

The most widely used treatment for fear and anxiety disorders has been extinction (clinically referred to as exposure therapy). In conditioned fear paradigms, extinction involves exposing a previously conditioned subject repeatedly to the fear-eliciting—but not inherently aversive—stimulus (conditional stimulus; CS) in the absence of the aversive stimulus (unconditional stimulus; US). This leads to gradual reduction in the emotional response to the CS (Pavlov, 1927). Typically, this experience is not incorporated into the original fear memory, but instead results in the formation of a new inhibitory memory (Barrett et al., 2003; Quirk and Mueller, 2008). Inhibitory memory formation is distinct from manipulations that are thought to alter the original memory by interfering with its reconsolidation (Dunsmoor et al., 2015). This difference is important, because extinction of the remaining fear memory generally leaves an organism vulnerable to return of fear through the passage of time (spontaneous recovery), CS exposure in a new context (renewal), or additional US exposure (reinstatement).

Monfils et al. (2009) devised an approach to fear attenuation that uses extinction training to manipulate the original memory during a period of memory destabilization (reconsolidation). They were able to use extinction to incorporate new information during reconsolidation by giving extinction training during a retrieval-induced labile period. This manipulation persistently attenuated fear responses in spontaneous recovery, renewal and reinstatement paradigms, implicating retrieval + extinction as a novel and non-invasive approach to memory updating.

Under normal conditions, reconsolidation updates memories by incorporating new contextual information into the memory trace (Jarome et al., 2015). Further evidence suggests that prediction error is necessary for reconsolidation interference to occur (Sevenster et al., 2012, 2013, 2014), but not sufficient (Sevenster et al., 2014). It still remains somewhat unclear, however, what other extinction conditions lead to memory updating. Recently, the emergence of new normative learning models that integrate seasoned learning models (e.g., Rescorla and Wagner, 1972; Pearce and Hall, 1980) with more complex principles of computational learning theory (e.g., Bayesian statistical inference) has shed new light on the field of learning and memory. Courville et al. (2003, 2005, 2006) were among the first to describe classical conditioning in terms of Bayesian inference and uncertainty. According to this statistical model, surprise due to events that deviate from expectations results in uncertainty and the need for new learning (Courville et al., 2006). Gershman and Niv (2012) combined these principles with reinforcement learning models proposed by Redish et al. (2007) and human rational categorization models proposed by Anderson (1991) to come up with a latent cause theory of classical conditioning (see Gershman et al., 2010 for a detailed comparison of these models). According to this theory, an animal combines its *a priori* beliefs about the structure of the world with its current observations (1) to make inferences about how CSs and USs are linked; and (2) to make predictions about possible future occurrences of a US. In regard to extinction learning, the theory posits that the subject discovers a new state of the world during extinction training—distinct from the state it experienced during fear memory acquisition—which warrants the formation of an entirely new memory (Redish et al., 2007; Gershman et al., 2010; Gershman and Niv, 2012).

In addition to the absence of the US evoking a prediction error during extinction, we posit that prediction errors could arise from parameters such as the time elapsed between the stimuli. Particularly, if the interval between CSs during extinction is fixed (i.e., occurring at regular, predictable intervals), then the inter-trial intervals (ITIs)—by evoking a predictable sort of “rhythm” to extinction training—may serve as an additional cue differentiating extinction from acquisition, which typically consists of fewer trials and fewer ITIs. As a result, fixed ITIs in extinction may lead to the inference of a new latent cause, and increase the probability of new learning rather than overwriting the original fear association. As such, we hypothesize that more fixed, less variable ITIs in extinction will lead to more eventual recovery of fear, as compared to more variable (i.e., random or unpredictable) ITIs. Accordingly, ITIs with greater variability may increase the likelihood of memory updating over new memory formation by preventing the inference of a new latent predictability cause. Integrating this idea with principles of the retrieval + extinction paradigm, we hypothesize that variable-ITI extinction administered during the reconsolidation window will produce optimal conditions for persistent fear attenuation. As such, rather than fostering conditions that would simply lead to stronger extinction (which would remain susceptible to the return of fear), we propose that the combination of destabilizing the memory with an isolated retrieval followed by extinction training with a variable ITI will promote memory updating.

**Figure 1:**
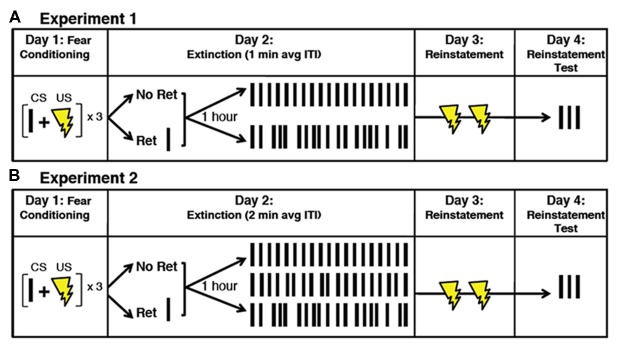
**Illustration of the experimental paradigm.** **(A)** Subjects received fear conditioning on Day 1, which consisted of three CS-US pairings. On Day 2, subjects received either a retrieval CS or no retrieval CS, followed 1 h later by extinction (19 total CSs) with either fixed ITIs or variable ITIs. Average ITIs were 1 min in Experiment 1 and 2 min in Experiment 2 min. **(B)** In Experiment 2 min, the ITI variability was divided into three different categories: no variability (fixed), small variability and large variability. On Day 3, subjects received two unpaired USs (reinstatement). On Day 4, subjects received three unpaired CSs as a probe for reinstatement of fear. CS, conditional stimulus; US, unconditional stimulus; ITI, inter trial interval; ret, retrieval; min, minute.

In the following experiments, we examined the interaction between memory destabilization via retrieval and variability of the extinction ITI on updating of a previously conditioned CS-US (tone-shock) association. We hypothesized that a variable ITI would result in less predictable CSs, and that such unpredictability would prompt extinction training that resembles the conditioning experience enough to promote memory updating over new memory formation. To test this, we gave fear-conditioned subjects either a retrieval CS or no retrieval CS, followed by extinction with either fixed ITIs or ITIs with some degree of random variability. We then tested reinstatement of the fear memory by re-exposing all subjects to the US and assessing the return of fear. In Experiment 1, we determined whether a variable (random) extinction ITI was more effective than a fixed extinction ITI at promoting reconsolidation updating using a 1-min mean extinction ITI. In Experiment 2, we tested the effect of further increasing the variability in ITI on reconsolidation updating by using an average ITI length of 2 min. This way, in addition to adding more random variability, we could also include a condition that involved the smaller amount of variability tested in Experiment 1, but over a longer time frame (see Figure 1 for full experimental design).

## Materials and Methods

### Subjects

Subjects were male Sprague-Dawley rats (250–300 g; Harlan Laboratories, Inc., Indianapolis, IN, USA). Sixty-four rats were used in Experiment 1 and 60 rats were used in Experiment 2. All procedures were conducted in compliance with the National Institute of Health Guide for the Care and Use of Experimental Animals and were approved by the University of Texas at Austin Institutional Animal Care and Use Committee. Rats were housed in pairs and maintained on a 12-h light/dark cycle with *ad libitum* availability of food and water.

### Apparatus and Stimuli

All behavioral procedures were conducted in standard conditioning chambers (Coulbourn Instruments) equipped with stainless-steel rod floors connected to shock generators. Each chamber was encased in an acoustic isolation box (Coulbourn Instruments) and illuminated by a red house light. Each chamber was wiped down with Windex between sessions.

Delivery of the stimuli was controlled by Freeze Frame software, and behavior was recorded using infrared digital cameras mounted on the ceiling of each chamber (Coulbourn Instruments). Each experiment used a tone (5 kHz, 80 dB) CS played for 20 s and a footshock (0.7 mA) US that lasted for 500 ms.

### Behavioral Procedures

#### Habituation

The day prior to fear conditioning, subjects were introduced to the context and allowed to explore for 10 min. The purpose of this exposure was to minimize fear conditioning to the context.

#### Fear Conditioning

On the day of fear conditioning, rats were habituated to the context for 3 min prior to receiving three 20-s presentations of the tone CS. Each CS co-terminated with a 0.7 mA footshock. For Experiment 1, the ITIs between CS presentations were 1 min, similar to the average ITI used subsequently during extinction. For Experiment 2, the ITIs during fear conditioning were 2 min. Rats remained in the conditioning chamber for 2 min after their last US presentation.

#### Retrieval and Extinction

##### Experiment 1

On the following day, subjects were placed back into the same context. Subjects were given 3 min to habituate and then received either extinction training alone (no retrieval condition), or a single retrieval CS followed 60 min later by extinction (retrieval condition). In between the retrieval CS and the extinction session in the retrieval condition, subjects were placed back into their home cages. Extinction consisted of 18 (retrieval condition) or 19 (no retrieval condition) CSs separated by either exactly 60 s (fixed ITI condition) or an interval that varied from 20 s to 100 s scaled to a normal distribution with a 60 s mean and a standard deviation of 30 s (variable ITI condition; see Table 1). The total number of CSs were the same in all groups, since the mean interval was 60 s in all conditions and subjects that did not receive a retrieval CS received one additional extinction CS. The total length of extinction (not including the retrieval portion) was 24 min for the extinction group (3 min habituation, 19 CSs, and 2 min post last CS) and 23 min for the retrieval + extinction group (3 min habituation, 18 CSs and 2 min post last CS).

**Table 1:** **Extinction ITI parameters**.

	ITI average (s)	Min ITI (s)	Max ITI (s)	SD	SD: mean ITI
**Experiment 1**
Not variable (fixed)	60	60	60	0	0
Variable	60	20	100	30	0.5
**Experiment 2**
Not variable (fixed)	120	120	120	0	0
“Small” variability	120	80	150	15	0.125
“Large” variability	120	20	240	60	0.5

*In Experiment 1, the average inter-trial interval (ITI) was 60 s. In one extinction condition, the ITI did not vary, thus the minimum (min) ITI and maximum (max) ITI were 60 s, and the standard deviation (SD) and SD: average ITI ratio was 0. In the other extinction condition, the ITIs were randomized and thus had a non-zero SD and SD: mean ITI ratio. In Experiment 2, the average ITI was 120 s. One extinction condition had a non-variable ITI, similar to Experiment 1. The range of ITIs in the “small” variability extinction condition was similar to that of the variable condition in Experiment 1. The SD: mean ITI ratio for “large” variability condition was the same as the variable condition from Experiment 1*.

##### Experiment 2

Similar to Experiment 1, on the day after fear conditioning, subjects were placed back into the same context, given 3 min to habituate followed by either extinction alone or an isolated retrieval CS. In the retrieval condition, subjects were transported back to their home cages for 60 min, then returned to the chamber for extinction. In this experiment, subjects were given one of three ITI conditions during extinction: exactly 120 s (fixed ITI condition), an interval that varied from 90 s to 150 s (small variability ITI; scaled to a normal distribution with a mean of 120 s and a standard deviation of 15 s), or an interval that varied from 20 s to 240 s (large ITI variability condition; scaled to a normal distribution with a mean of 120 s and a standard deviation of 60 s; see Table 1). Thus the small variability condition used a *range* of intervals similar to Experiment 1, whereas the large variability condition resulted in a SD: mean ITI ratio similar to Experiment 1. This way, we could determine whether the variability effect we observed in Experiment 1 could be explained by the absolute deviation of ITIs from the mean or if the effect was driven by the relationship between the standard deviation of the ITIs and the mean ITI. The number of CSs was the same in all groups, since the mean interval was 120 s in all conditions and subjects that did not receive a retrieval CS received one additional extinction CS. The total length of extinction (not including the retrieval portion) was 43 min for the extinction group (3 min habituation, 19 CSs, and 2 min post last CS) and 41 min for the retrieval + extinction group (3 min habituation, 18 CSs, and 2 min post last CS).

#### Reinstatement

Twenty-four hours after extinction, subjects were placed back into the chambers, allowed to habituate for 5 min, followed by two exposures to the US alone. US presentations were separated by 1 min in Experiment 1 and 2 min in Experiment 2. Subjects remained in the context for 5 min following the last US before being put back into their home cages. The following day, subjects were placed back into the context and given three presentation of the CS alone to probe for reinstatement-induced return of fear. The timing between the CSs during the reinstatement probe was 1 min (fixed) for Experiment 1 and 2 min for Experiment 2.

### Scoring—Freezing

Freezing was defined as the cessation of movement while in the crouched position, excluding breathing, whisker twitching and resting/sleeping. The total number of seconds spent freezing during the 20 s prior to the CS (pre-CS) as well during the 20-s CS is expressed as a percentage of pre-CS/CS duration. All scoring was performed off-line manually by an observer blind to the experimental conditions.

### Statistical Analysis

Statistical analyses were carried out using PASW Statistics software version 18.0 and R Statistical Computing software. Mixed factor ANOVAs with the CS cue as a repeated measure, and retrieval group and variability group membership as between subject factors. Where appropriate, *post hoc* tests were performed with Tukey’s honestly significant difference mean comparison. Percent freezing at the end of extinction is expressed as the mean percentage across the final three CSs during extinction training; percent freezing during reinstatement tests is expressed as the mean percentage across three CS presentations.

## Results

### Experiment 1: Variable Extinction ITI Leads to a More Persistent Attenuation of Freezing

To examine the effect of a fixed vs. variable ITI, we used a 2 × 2 factorial design, whereby fear-conditioned subjects received either extinction only (i.e., no retrieval CS) or a retrieval CS followed 60 min later by extinction with either fixed or variable (normally-distributed) ITIs. The day after extinction, all subjects were re-exposed to the US only and probed the following day for CS-induced return of fear (Figure 1). All groups showed a significant within-subjects reduction in freezing during extinction, as revealed by a repeated-measures analysis of variance (ANOVA) using the average freezing during the first three and last three CSs as the repeated factor and the retrieval condition and the ITI variability condition as the between-subjects factors (*F*_(1,60)_ = 187.2, *P* = 0.001, $\eta_{\text{p}}^{2}$ = 0.757).

**Figure 2:**
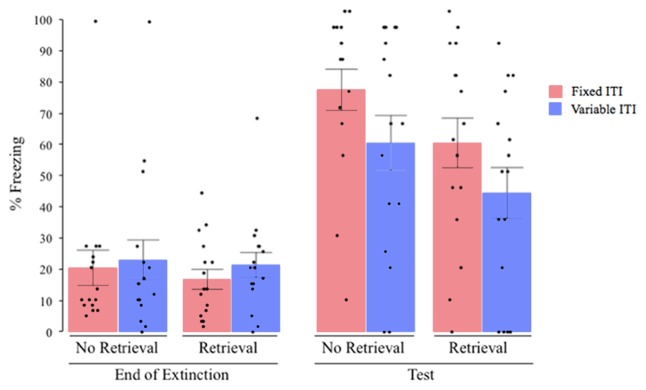
**Percent freezing at the end of extinction and post reinstatement for Experiment 1 (1-min average ITI).** All groups showed a significant increase in freezing from the end of extinction to post-reinstatement test, with significant main effects of ITI variability and retrieval condition on the overall amount of freezing at test, with no interaction between them. ITI, inter-trial interval; black dots represent individual data points.

We then assessed the return of fear via reinstatement. A univariate ANOVA of freezing at test with the retrieval condition and the ITI variability condition as the between subjects factors and co-varying for freezing at the end of extinction revealed significant main effects of both retrieval (*F*_(1,59)_ = 3.993, *P* = 0.05, $\eta_{\text{p}}^{2}$ = 0.063) and ITI variability (*F*_(1,59)_ = 5.189, *P* = 0.026, $\eta_{\text{p}}^{2}$ = 0.081), with no significant effect of terminal extinction freezing (*F*_(1,59)_ = 3.118, *P* = 0.083, $\eta_{\text{p}}^{2}$ = 0.050) and no significant interaction (*F*_(1,59)_ = 0.00, *P* = 0.988, $\eta_{\text{p}}^{2}$ = 0.001. *Post hoc* analysis revealed (see Figure 2) that, overall, on average, subjects that received extinction training with variable ITIs showed lower post-reinstatement freezing than subjects that received extinction with fixed ITIs (*post hoc* comparison between fixed, vs. variable; collapsing across extinction group). Likewise, subjects that received a retrieval cue 1 h before extinction showed lower post-reinstatement freezing than subjects that did not receive the retrieval cue (*post hoc* comparison between extinction vs. retrieval + extinction groups; collapsing across ITI type). Subjects that received both retrieval cue and a variable extinction ITI showed the lowest post-reinstatement freezing, but interestingly, all groups showed significant post-reinstatement return of fear, as revealed by an increase in freezing from the end of extinction to post-reinstatement using paired-samples *t*-tests (all *P*’s < 0.05).

The results indicate that receiving the retrieval CS 1 h before extinction resulted in less return of fear after reinstatement than groups that received extinction alone, without influencing the degree of extinction within-session (Figure 4). Also, receiving extinction with a variable ITI resulted in less return of fear. The lack of interaction between the two factors suggests that both retrieval and variable ITIs operated independently to attenuate fear.

### Experiment 2: Increasing Variability in Extinction ITI Is Associated with Reduced Freezing

Once we determined that introducing variability to 1-min average extinction ITIs helped attenuate the fear memory, we wanted to broaden our understanding of how variability affects persistent fear reduction. We tested the prediction that increased variability would reduce the return of fear by introducing more variability over a longer extinction session and directly comparing it to a low variability condition. For this we extended the extinction session such that the average ITI was 2 min to allow for more random variability. We used a 3 × 2 factorial design, whereby fear-conditioned subjects received either extinction only (i.e., no retrieval CS) or a retrieval CS followed 60 min later by extinction with no variability (fixed ITI), low variability (similar to the amount of variability present in Experiment 1), or high variability in the ITIs. In comparison to Experiment 1, the overall variability in length of ITIs was larger in Experiment 2, in terms of the range of possible time intervals.

The day after extinction, all subjects were re-exposed to the US only and probed the following day for CS-induced return of fear (Figure 1). Again, all groups showed a significant within-subjects reduction in freezing during extinction training, as revealed by a repeated-measures analysis of ANOVA using the average freezing during the first three and last three CSs as the repeated factor and the retrieval condition and the ITI variability condition as the between-subjects factors (*F*_(1,51)_ = 199.8, *P* = 0.000, $\eta_{\text{p}}^{2}$ = 0.785).

**Figure 3:**
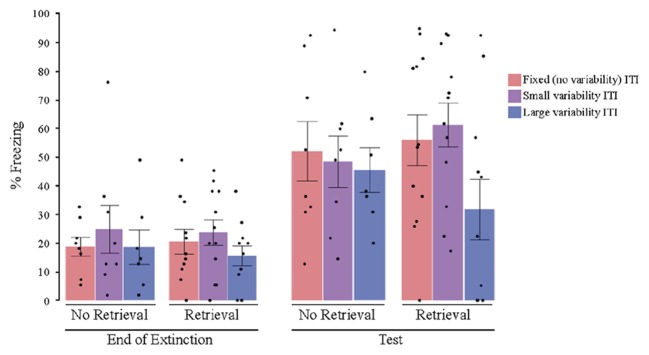
**Percent freezing at the end of extinction and post reinstatement for Experiment 2 (2-min average ITI).** The results showed a variability by retrieval condition interaction. The group that received both a retrieval CS and highly variable ITIs showed less post reinstatement freezing, and also did not show a significant increase in freezing from the end of extinction to post-reinstatement test. All other groups showed significant increases in freezing from the end of extinction to test, with no significant differences among the groups. ITI, inter-trial interval; black dots represent individual data points.

After controlling for post-extinction freezing—which was a significant covariate in this case (*F*_(1,50)_ = 5.560, *P* = 0.038, $\eta_{\text{p}}^{2}$ = 0.084)—a univariate ANOVA with the retrieval condition and the ITI variability condition as the between subjects factors revealed no significant main effects for either retrieval (*F*_(1,50)_ = 0.014, *P* = 0.907, $\eta_{\text{p}}^{2}$ = 0.001) or ITI variability (*F*_(2,50)_ = 1.252, *P* = 0.295, $\eta_{\text{p}}^{2}$ = 0.048). However, there was a significant interaction between retrieval and ITI variability (*F*_(2,50)_ = 3.261, *P* = 0.047, $\eta_{\text{p}}^{2}$ = 0.115). *Post hoc* planned comparisons showed that the high variability group exhibited less post-reinstatement freezing than the fixed and low variability groups in the retrieval condition (Figure 3; *t*_(32)_ = 2.696, *P* = 0.011, $\eta_{\text{p}}^{2}$ = 0.144), but not in the no retrieval condition (*t*_(32)_ = −0.229, *P* = 0.821, $\eta_{\text{p}}^{2}$ = 0.028). Cohen’s effect size value for the retrieval condition planned comparison (*d* = 0.953) suggests a high practical significance. A direct comparison of the high variability no retrieval vs. retrieval groups showed a trend toward a significant difference (*t*_(17)_ = 1.839, *p* = 0.083, *d* = 0.878).

### Pre-CS Freezing

**Figure 4:**
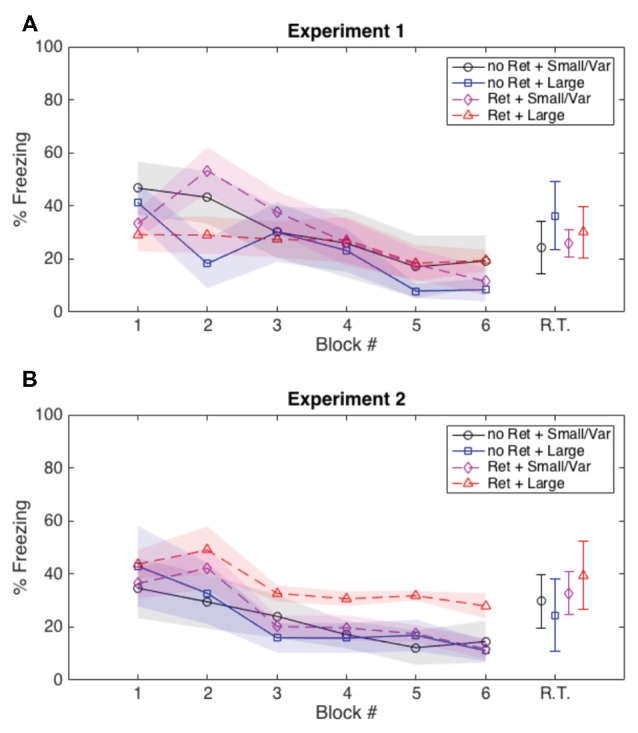
**Average freezing during the 20 s prior to each CS presentation during extinction and post-reinstatement test. (A)** In Experiment 1 there were no group differences in pre-CS freezing during extinction or prior to the reinstatement test. **(B)** In Experiment 2, all groups showed similar pre-CS freezing during the first half of extinction, but the group that received the retrieval CS and highly variable ITIs showed more preCS freezing during the second half of extinction than the other groups, presumably due to uncertainty about when the CS would appear. These group differences in pre-CS freezing did not persist through reinstatement, and thus cannot be attributed to generalized freezing rather than a response to ITI variability. R.T., retrieval test; shaded areas and error bars: ±1 standard error of the mean; “Ret”, retrieval; “Large”, large variable ITI; “Small/Var”, small variable and fixed ITI.

Both Experiments 1 and 2 suggest that variability in the ITIs during extinction leads to greater attenuation of fear memory. With these data, we cannot directly measure how ITI variability influences the “predictability” of the CS; however, we were able to gain some insight indirectly by analyzing the amount of freezing subjects displayed in the 20 s preceding each extinction CS. In Experiment 1 (in which we saw a more persistent reduction of fear with a variable ITI, but all groups showed some degree of return of fear), we did not see any group differences in Pre-CS freezing. However, in Experiment 2 (in which the group that received both a retrieval CS and a largely variable ITI showed no significant return of fear), Pre-CS freezing was the same in all groups in the first half of extinction, but Pre-CS freezing for the retrieval + largely variable ITI group remained higher than the other groups as extinction progressed (Figure 4). We argue that a subject that freezes during the Pre-CS interval is uncertain when another CS will occur. Conversely, a subject that does not freeze during the Pre-CS interval has a clearer expectation about when a CS is (and is not) going to occur. The retrieval + large variability ITI group froze more during the Pre-CS intervals than any other group, and also had the lowest amount of fear reinstatement. This suggests that the combination of memory destabilization via a retrieval CS and largely variable ITIs during extinction evoked greater freezing during the Pre-CS interval, which also resulted in greater attenuation of fear.

## Discussion

Behavioral interference via extinction training during a post-retrieval reconsolidation window has been shown to persistently attenuate fear responses (Monfils et al., 2009). New normative learning models posit that during extinction training, animals may infer a new state that is different from that in acquisition training, and thereby form (and update) a new association or memory, rather than apply the experiences during extinction to updating the old acquisition association (Redish et al., 2007; Gershman et al., 2010; Gershman and Niv, 2012). This could explain why typical extinction paradigms result in incomplete attenuation of fear, and eventual recurrence of fear of the CS. Drawing support from these models, we hypothesized that exposing subjects to random, unpredictable CSs during extinction would lessen the coherence of the extinction experiences, and therefore the degree to which subjects infer a new state, thus promoting more memory updating. Our results indeed supported this proposal, as subjects who received a retrieval cue followed by extinction with a variable ITI showed more persistent reduction of fear than subjects who received fixed extinction ITIs.

In Experiment 1, with an average ITI of 1 min in extinction, we observed main effects of the two treatment conditions: subjects who received an isolated retrieval CS prior to extinction showed less post-reinstatement return of fear on average than subjects who did not receive the retrieval cue; subjects who received variable extinction ITIs showed less post-reinstatement return of fear than subjects who received fixed ITIs. There were no interactions and no differences in Pre-CS freezing, suggesting that memory destabilization via an isolated retrieval CS variable extinction ITIs each led to diminished reinstatement of fear regardless of the other condition. In Experiment 2, we repeated the procedures of Experiment 1, however with 2-min ITIs on average, and testing two levels of variability of ITIs. In this experiment, we observed a significant interaction between the retrieval and variability conditions—variability in ITIs increased fear attenuation only when the memory was already destabilized via an isolated retrieval CS, and only in the large variability condition. Unlike for Experiment 1, when we extended the average ITI from 1 to 2 min, neither retrieval-induced memory destabilization nor variable extinction ITIs were sufficient conditions for persistent fear attenuation; rather, both were necessary for maximal fear memory attenuation. This result is in line with our previous work, in which we showed that extinction with a larger (3 min) and variable ITI presented after an isolated retrieval prevented the return of fear (Monfils et al., 2009). We now extend these findings to suggest that the variable and larger ITI are necessary (we did not explicitly test the effect of ITI length or variability in Monfils et al., 2009).

In Experiment 2, the group that received a retrieval CS + large variability extinction ITI (which is also the only group in either experiment that showed no increase whatsoever in freezing from post-extinction to post-reinstatement) froze more in the 20 s preceding each CS during the second half of extinction. This suggests that the subjects in this group were more uncertain than any other group about when an aversive stimulus could occur, and thus froze more during the ITI. Since we did not observe the same group differences in freezing during the CSs (which indicates anticipation for USs), we can reasonably assume that all groups experienced the same reduction in expectation for a paired CS-US. Consequently, by using a variable ITI and thus manipulating subjects’ ability to anticipate any stimulus at all, we effectively mediated the amount of memory updating induced by extinction.

Evidence suggests that stress can be detrimental to memory reconsolidation (Maroun and Akirav, 2008; Wang et al., 2009; Maren and Holmes, 2016), so perhaps one explanation for this finding lies in the possibility that by reducing a subject’s ability to predict when the CS occurs during extinction, we generally increased the amount of stress the subject experienced during a time period critical for reconsolidation of the original memory elicited by the isolated retrieval CS. New evidence supports that the larger the discrepancy between expected and observed outcomes, the more likely the animal is to infer a new state of the world, and thus is more likely to form a new memory for the experience rather than update an old one (Redish et al., 2007; Gershman et al., 2010; Gershman and Niv, 2012). We observed new learning when predictability of the CS during extinction was high (as evidenced by higher return of fear in the fixed ITI conditions). We posit that because CS-US presentation during fear conditioning was surprising, an animal may be less likely to infer a new latent cause during extinction with variable ITIs because random CS-only presentation during extinction is also surprising. In other words, the animal learns to expect unpredictability in stimulus timing; thus, when timing becomes predictable, a new latent cause emerges leading to new memory formation. Still, our result that the pre-CS freezing remains greater in the retrieval + extinction group with the large ITI variability is surprising. How could such a simple manipulation lead to increased uncertainty? Effectively, reconsolidation manipulations do not typically show an effect in the short term (e.g., Nader et al., 2000a; Ponnusamy et al., 2016), and only surface during the long-term memory test. The present results suggest that while behavioral evidence for reconsolidation influences may be “unmasked” at a later time point (e.g., LTM and beyond), other, perhaps less tangible, changes do occur. Even though they may not *a priori* seem directly related to a reconsolidation updating effect, they seem unlikely to simply be occurring by chance alone (only one group out of 10 showed increased pre-CS freezing, and that same group is the only only that did not show significant reinstatement). We propose that the increased variability occurring in the context of a destabilized memory increases uncertainty, thereby promoting the updating of a memory rather than the creation of a new (as is generally the case with extinction learning; Gershman and Niv, 2012).

Though we understand that reactivated memories change, situations and mechanisms that instigate memory updating remain subject to different interpretations (Gisquet-Verrier et al., 2015). Could attenuated return of fear following post-retrieval extinction be explained by a potential effect of spaced vs. massed extinction, or augmented/boosted extinction? We do not believe this could be the case. Recent studies have indeed shown that post-retrieval extinction engages mechanisms that are distinct from “standard” extinction (Monfils et al., 2009; Clem and Huganir, 2010; Rao-Ruiz et al., 2011; Tedesco et al., 2014; Lee et al., 2016). One explanation of memory updating is that an experience that both evokes a memory for *and* greatly resembles a previous experience (i.e., possess the same latent causes) is likely to be integrated with the previous memory. Conversely, an experience that evokes a memory but also possess unique elements may qualify as a new situation that is due to a different underlying latent cause and thus requires new encoding and separate consolidation. In our experiments, extinction training administered during the post-retrieval reconsolidation window resulted in maximal fear memory attenuation when extinction consisted of CSs presented at variable (random) intervals, rather than fixed (predictable) intervals. We posit that this randomness created a situation whereby extinction training maximally resembled the unpredictable nature of the fear conditioning to which the rats had been exposed previously, and thus promoted memory updating over new memory formation. Though these results are promising, further translational investigation is needed to determine whether unpredictability and random exposure increases the efficacy of current clinical treatments for fear and anxiety disorders.

## Author Contributions

AA ran all experiments. AA and MHM designed, analyzed and interpreted all experiments, and wrote the first draft of the manuscript. YN and LKC contributed to experiment design. LKC, YN and FG-L contributed to analyses and interpretation, as well as edited the manuscript and approved final version.

## Conflict of Interest Statement

The authors declare that the research was conducted in the absence of any commercial or financial relationships that could be construed as a potential conflict of interest.
